# Telomere-protecting protein 1 promotes gastric cancer cell metastasis *via* enhancing endoplasmic reticulum stress

**DOI:** 10.1016/j.jbc.2025.110998

**Published:** 2025-12-01

**Authors:** Zhifu Gui, Leimin Qian, Lin Gao, Zhihua Feng, Xiao Nie, Zhuoqi Xuan, Wei Guo, Siyuan Zhang, Kun Zhang, Ying Xu, Wei Zhao

**Affiliations:** 1 Department of General Surgery, Jiangyin Clinical College of Xuzhou Medical University, Jiangyin, China; 2 Department of General Surgery, Jiangyin People's Hospital Affiliated to Nantong University, Jiangyin, China; 3 Department of General Surgery, Jiangyin Clinical Medical College of Jiangsu University, Jiangyin, China; 4 Department of Pathology, Jiangyin Clinical College of Xuzhou Medical University, Jiangyin, China; 5 School of Laboratory Medicine, Chengdu Medical College, Chengdu, China; 6 The Second Affiliated Hospital of Chengdu Medical College, China National Nuclear Corporation 416 Hospital, Chengdu, China; 7 Department of Oncology, Affiliated Cancer Hospital of Chengdu Medical College, Chengdu, China; 8 School of Biological Sciences and Technology, Chengdu Medical College, Chengdu, China; 9 School of Clinical Medicine, The First Affiliated Hospital, Chengdu Medical College, Chengdu, China

**Keywords:** endoplasmic reticulum stress, gastric cancer, *H*. *pylori*, metastasis, telomere-protecting protein 1

## Abstract

Gastric cancer (GC) is one of major global cancers that is highly heterogeneous and has a poor prognosis, especially in cases associated with *Helicobacter pylori* (*H*. *pylori*) infection. *H*. *pylori* promote metastasis *via* mechanisms like endoplasmic reticulum stress (ERS). Telomere-protecting protein 1 (TPP1), a telomere-protecting protein, is overexpressed in GC and linked to poor outcomes. This study investigated TPP1's role in *H*. *pylori*-induced ERS and its implications for GC metastasis.We analyzed TPP1 expression using both Starbase data and clinical samples. Functional assays (*e*.*g*., migration, invasion, wound healing) were performed in GC cell lines with TPP1 knockdown or overexpression. The interaction between TPP1 and ERS-related proteins was assessed by immunoprecipitation and immunofluorescence. The role of TPP1 in GC metastasis was further validated in a xenograft mouse model. TPP1 was upregulated in GC tissues and cell lines, correlating with poor prognosis. TPP1 knockdown inhibited GC cell metastasis but not proliferation, while TPP1 overexpression enhanced metastasis. *H*. *pylori* enhanced TPP1 expression by stabilizing Enhancer of Zeste Homolog 1, which in turn binds to the TPP1 promoter. TPP1 interacted with 78 kDa glucose-regulated protein, disrupting its binding to PKR-like ER kinase and activating ERS. Blocking ERS reversed the pro-metastatic effects of TPP1 overexpression. *In vivo*, TPP1 knockdown significantly reduced GC metastasis in nude mice xenograft model. TPP1, induced by *H*. *pylori*, promoted GC metastasis by enhancing ERS *via* interaction with glucose-regulated protein. Targeting the TPP1/ERS axis may offer a novel therapeutic strategy for GC.

Gastric cancer (GC) is a prevalent cancer worldwide, and the incidence of GC ranks fifth among malignant tumors. In 2022, there were approximately 969,000 new cases of GC globally, with the highest incidence in the Asian region, accounting for 75.7% of global cases (1). GC is a disease with high molecular and phenotypic heterogeneity, characterized by poor clinical outcomes, strong invasiveness and metastatic ability, and short survival duration. Surgical resection is the main treatment for GC, and peritoneal metastasis is the primary factor affecting the prognosis of GC patients (2). Currently, there are no specific early diagnostic biomarkers or detection methods for GC. As a result, more than 70% of GC patients are diagnosed at an advanced stage, and about 40% of patients have metastasis to varying degrees. Among these patients, only about 5% can survive for 5 years (3, 4). Moreover, even patients who undergo radical resection for GC without distant metastasis before surgery may experience recurrence and metastasis in nearly 50% of cases after surgery (5). Therefore, elucidating the possible mechanisms of GC metastasis is of great significance for identifying new targets for precision treatment of GC patients and effectively improving their prognosis.

*Helicobacter pylori* is a core pathogenic factor in the development of GC, with its mechanisms of action involving multi-level regulation at the molecular, cellular, and microenvironmental levels. Approximately 50% of the global population is infected with this bacterium, and infected individuals have a 2- to 11-fold increased risk of GC. However, only 1 to 3% of infected individuals develop GC, suggesting a synergistic effect of host genetic susceptibility and environmental factors such as smoking (6, 7). *H*. *pylori* directly damage the gastric mucosa through virulence factors such as the CagA protein. The East Asian type of CagA, with its stronger tyrosine phosphorylation ability, can significantly interfere with host cell signaling, leading to a higher risk of carcinogenesis (8, 9). *H*. *pylori* infection can activate the epithelial-mesenchymal transition (EMT) pathway, promoting tumor invasiveness through the TGF-β1/Smad2 signaling cascade and remodeling the tumor microenvironment to enhance immune evasion (10). *H*. *pylori* infection is claimed to induce endoplasmic reticulum stress (ERS) in gastric epithelial cells through virulence factors such as VacA, activating the unfolded protein response (UPR) and leading to the overexpression of key proteins such as HSPA5 and XBP1, thereby promoting GC metastasis (11).

ERS is a key regulatory factor in the tumor metastasis microenvironment, driving tumor cell invasion, migration, and distant colonization through the abnormal activation of the UPR signaling pathway (12). Research indicates that ERS can regulate the expression of tumor metastasis-related genes through core sensors (13), such as glucose regulatory protein 78 (GRP78) (14) and protein kinase R-like endoplasmic reticulum kinase (PERK) (15). For example, the nuclear translocation of ATF6 can induce translational repression of EMT-related molecules such as HMGA2, while the IRE1-XBP1 pathway activated by ERS promotes tumor cell survival and vasculogenic mimicry formation under nutrient-deprived conditions (16, 17). In addition, the metastatic tendency of chromosomal instability tumors is enhanced by ERS through the activation of the cGAS-STING pathway, a process that is particularly prominent in an immunocompetent microenvironment, suggesting a synergistic effect between ERS and tumor immune evasion (18). Therefore, in-depth exploration of the regulatory mechanisms of ERS induced by *H*. *pylori* in GC cells is conducive to revealing its metastatic mechanisms.

TPP1 (telomere-protecting protein 1,also name as TINT1/PIP1/PTOP1) is a core component of the shelterin complex, which protects telomeres and exerts multiple functions through its N-terminal oligonucleotide/oligosaccharide-binding (OB) domain (19, 20). TPP1 forms a stable heterodimer with POT1, and together with the OB1/OB2 domains of POT1, it binds to single-stranded telomeric DNA, effectively unwinding the G4 quadruplex structure and maintaining telomere stability (21, 22). Recent studies have shown that TPP1 is abnormally overexpressed in a variety of tumors (such as esophageal cancer, cervical cancer, and melanoma), promoting telomere elongation through the recruitment of telomerase and maintaining the replicative immortality of cancer cells (23, 24, 25). In GC, TPP1 has been identified as one of the key factors for predicting the progression and prognosis of GC (26). However, the specific regulatory mechanisms of TPP1 in GC are still unclear. This study explores the regulatory mechanisms of TPP1 on ERS to elucidate its critical role in GC and provides a potential molecular target for clinical GC treatments.

## Results

### TPP1 was markedly upregulated in GC tissues with the indication of a poor prognosis

**Figure 1:**
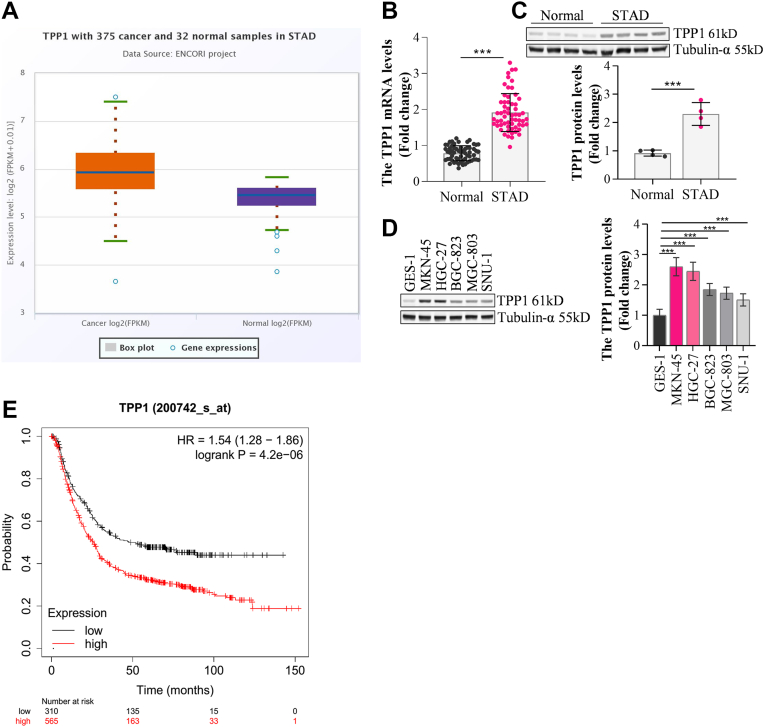
**TPP1 was upregulated in GC tissues, and it indicates a poor prognosis**. *A*, the expression of TPP1 in stomach adenocarcinoma (STAD) tissues was analyzed by Starbase. *B*, the expression of TPP1 in GC tissues was detected by RT-qPCR (N = 60, Normal refers to Adjacent tissues). *C*, the expression of TPP1 in GC tissues was detected by Western blot, and densitometry analysis was performed (N = 4, Normal refers to Adjacent tissues). *D*, the expression of TPP1 in normal cells and GC cells was detected by Western blot, and densitometry analysis was performed. *E*, The relationship between TPP1 and GC prognosis was analyzed by Kaplan-Meier Plotter (∗*p* < 0.05, ∗∗*p* < 0.01, ∗∗∗*p* < 0.001 indicate statistically significant differences. Ns indicates no significant difference).

According to Starbase analysis, TPP1 expressions were materially higher in stomach adenocarcinoma tissues comparing to normal tissues (Fig. 1*A*). Additionally, in clinical samples of GC tissues, TPP1 was sharply upregulated comparing to normal tissues (Fig. 1, *B* and *C*). Furthermore, comparing to GES-1 cells, expressions of TPP1 were notably enhanced in GC cell lines (MKN-45, HGC-27, BGC-823, MGC-803, and SNU-1 cells), among which highest expressions of TPP1 were observed in MKN-45 and HGC-27 cells (Fig. 1*D*). According to KM plotter analysis, high expressions of TPP1 were correlated with poor prognosis in GC patients (Fig. 1*E*).

### TPP1 knockdown suppressed GC cell metastasis but not proliferation

**Figure 2:**
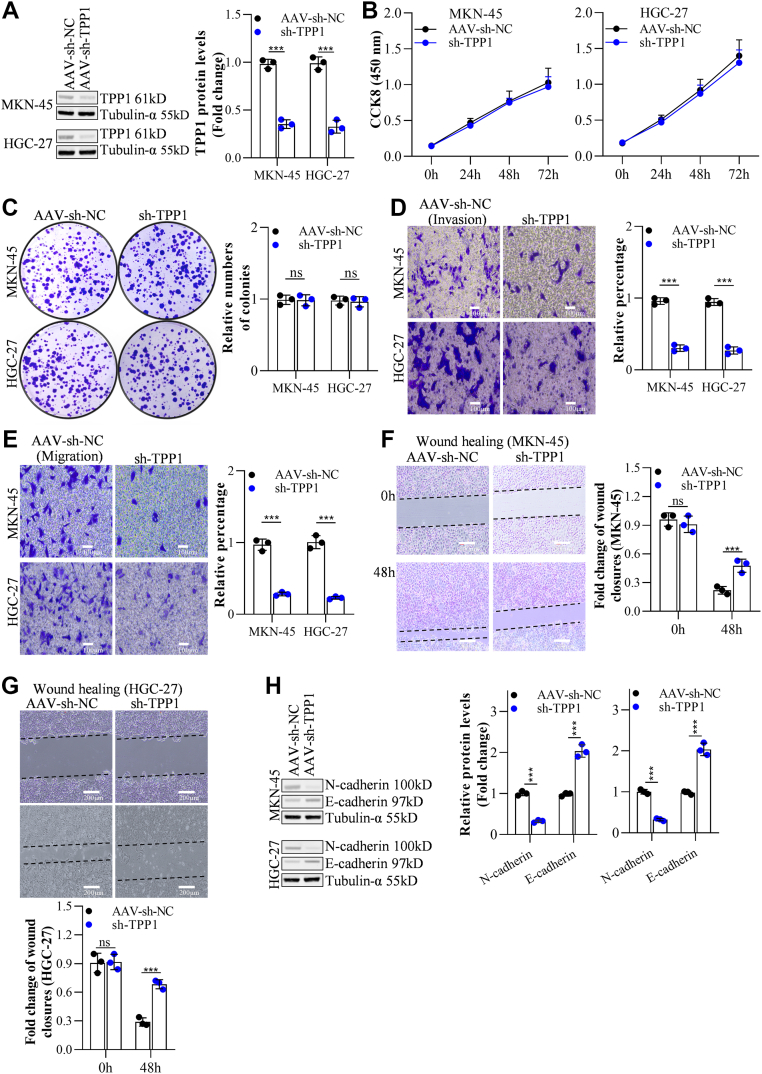
**TPP1 knockdown suppressed GC cell metastasis but not proliferation *in vitro***. *A*, the knockdown effect of TPP1 in GC cells was detected by Western blot (72 h post-infection with AAVs). *B*, the impact of TPP1 knockdown on the proliferation of GC cells was assessed by CCK-8 assay. *C*, the effect of TPP1 knockdown on the proliferation of GC cells was examined by colony formation assay. *D* and *E*, the influence of TPP1 knockdown on the invasion and migration of GC cells was evaluated by Transwell invasion and migration assays. *F* and *G*, the effect of TPP1 knockdown on the migration of GC cells was analyzed by wound healing assay. *H*, the impact of TPP1 knockdown on the expression of invasion and migration markers N-cadherin and E-cadherin was detected by Western blot. (∗*p* < 0.05, ∗∗*p* < 0.01, ∗∗∗*p* < 0.001 indicate statistically significant differences. Ns indicates no significant difference).

TPP1 was silenced in MKN-45 (decreased to 0.35 compared with control) and HGC-27 (decreased to 0.32 compared with control) cells to explore its function in GC cell metastasis and proliferation, knockdown efficacies of which were shown in Figure 2*A*. No dramatical differences were observed on cell viabilities (Fig. 2*B*) and relative numbers of colonies (Fig. 2*C*) in MKN-45 or HGC-27 cells following the knockdown of TPP1. However, silencing TPP1 strikingly decreased number of migrated (by 30.33 and 27.0% in MKN-45 and HGC-27, respectively) and invaded (by 28.45 and 23.24% in MKN-45 and HGC-27, respectively) GC cells (Fig. 2, *D* and *E*), accompanied by considerably enlarged fold change of wound closures (by 2.17 and 2.66 fold in MKN-45 and HGC-27, respectively) (Fig. 2, *F* and *G*). Furthermore, in TPP1-silenced MKN-45 or HGC-27 cells, N-cadherin expressions were noticeably diminished, while E-cadherin levels were largely raised, implying an inhibition of EMT progression as previous reports (27, 28) (Fig. 2*H*). Consistently, decreased TPP1 reduces MKN-45 or HGC-27 cells lung, liver and lymph node metastasis incidence *in vivo* (Supplementary Fig. S1).

### TPP1 overexpression enhanced GC cell metastasis but not proliferation

**Figure 3:**
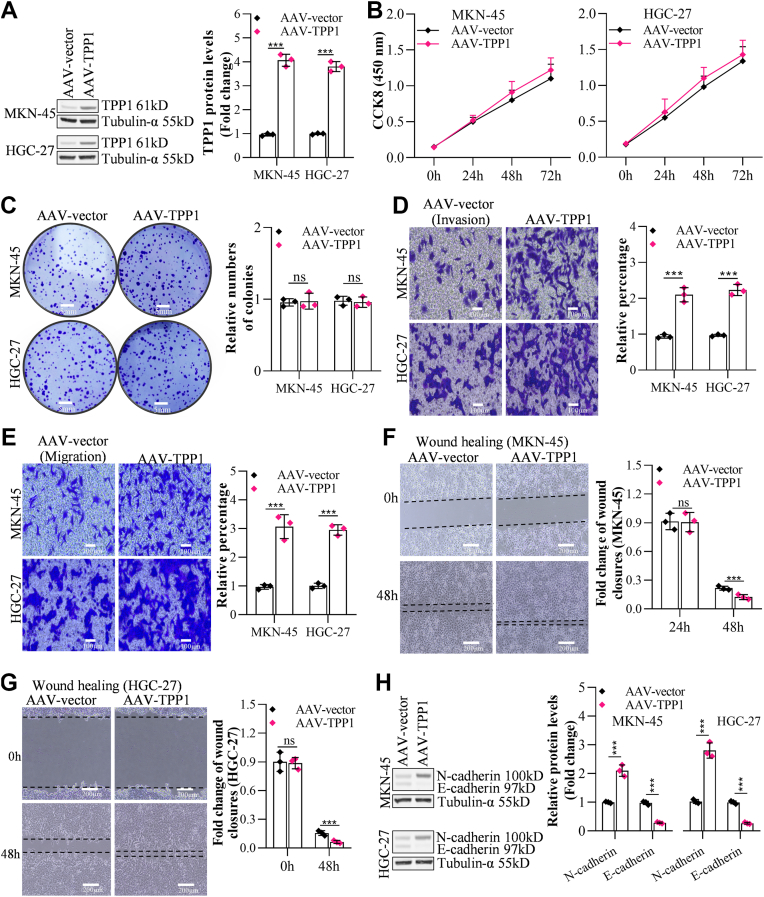
**TPP1 overexpression promoted GC cell metastasis but not proliferation *in vitro***. *A*, the effect of TPP1 overexpression in GC cells was detected by Western blot. *B*, the impact of TPP1 overexpression on the proliferation of GC cells was detected by CCK-8 assay. *C*, the effect of TPP1 overexpression on the proliferation of GC cells was detected by colony formation assay. *D* and *E*, the effects of TPP1 overexpression on the invasion and migration of GC cells were detected by Transwell invasion and migration assays. *F* and *G*, the impact of TPP1 overexpression on the migration of GC cells was detected by wound healing analysis. *H*, the effects of TPP1 overexpression on the expression of invasion and migration markers N-cadherin and E-cadherin were detected by Western blot. (∗*p* < 0.05, ∗∗*p* < 0.01, ∗∗∗*p* < 0.001 indicate statistically significant differences. Ns indicate no significant difference).

Subsequently, TPP1 was overexpressed in MKN-45 (4.67 fold compared with control) and HGC-27 (3.82 fold compared with control) cells to validate its function in GC cell metastasis and proliferation, overexpression efficacies of which were verified in Figure 3*A*. Cell viabilities (Fig. 3*B*) and relative numbers of colonies (Fig. 3*C*) in MKN-45 or HGC-27 cells were mildly altered after the overexpression of TPP1. However, numbers of migrated (by 2.22 and 2.23 fold in MKN-45 and HGC-27, respectively) and invaded (by 3.67 and 2.95 fold in MKN-45 and HGC-27, respectively) cells were perceptibly increased by overexpressing TPP1 (Fig. 3, *D* and *E*), along with markedly diminished fold change of wound closures (by 43.91 and 59.73% in MKN-45 and HGC-27, respectively) (Fig. 3, *F* and *G*). Moreover, in TPP1-overexpressed MKN-45 or HGC-27 cells, N-cadherin was appreciably upregulated, while E-cadherin were largely downregulated, hinting an activation of EMT progression (Fig. 3*H*).

### *H*. *pylori* increased TPP1 expressions by recruiting enhancer of zeste homolog 1 (EZH11) to TPP1 promoter

**Figure 4:**
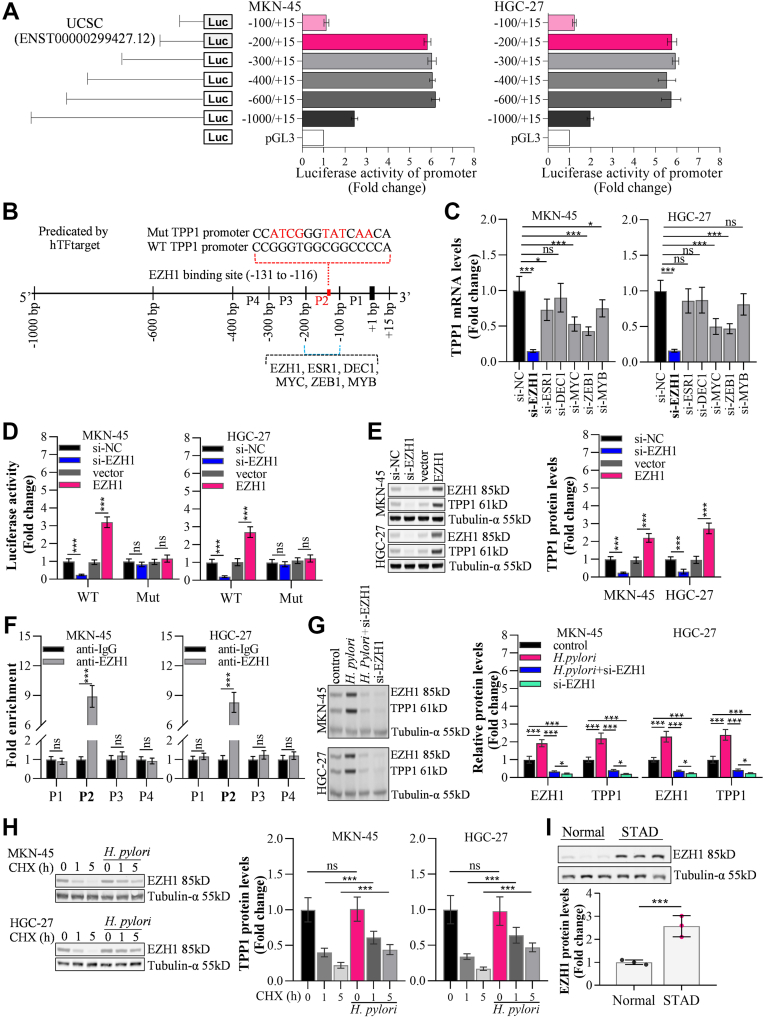
***H*. *pylori* increased TPP1 expression by recruiting EZH11 to TPP1 promoter**. *A*, the pGL3-basic plasmid system and its kit from Promega were used to detect the changes in luciferase activity of different regions of the TPP1 promoter, and it was found that the region between −200 bp and −100 bp was essential for the activation of the TPP1 promoter, while the region between −1000 bp and −600 bp may block the activation of the TPP1 promoter. *B*, the region between −200 bp and −100 bp was found to bind to transcription factors EZH1, ESR1, DEC1, MYC, ZEB1, and MYB through the online analysis tool hTFtarget. *C*, RT-qPCR detection revealed that the knockdown of the transcription factor EZH1 most significantly altered the expression of TPP1 mRNA. *D*, the pGL3-Basic plasmid system and its kit from Promega were used to detect that the mutation of the EZH1 binding site in the TPP1 promoter led to the failure of EZH1 to effectively regulate the luciferase activity of the TPP1 promoter (−200, +15). (*E*) Western blot was used to detect the effects of EZH1 knockdown or overexpression on the expression of the TPP1 protein. *F*, ChIP-qPCR was used to detect the binding of EZH1 to the TPP1 promoter (P1, P2, P3, and P4 represent pairs of primers for different regions). *G*, western blot revealed that *H*. *pylori* could promote the expression of EZH1 and TPP1 proteins, but the knockdown of EZH1 could reverse the promoting effect of *H*. *pylori* on the expression of the TPP1 protein. *H*, western blot revealed that *H*. *pylori* could promote the degradation of EZH1 protein under CHX treatment. (∗*p* < 0.05, ∗∗*p* < 0.01, ∗∗∗*p* < 0.001 indicate statistically significant differences. Ns indicate no significant difference).

To explore potential upstream regulatory mechanism for TPP1 in GC cells, the pGL3-Basic plasmid system and its kit from Promega were used to detect changes in luciferase activity in different regions of the TPP1 promoter. It was found that the region between −200 bp and −100 bp was essential for the activation of the TPP1 promoter. The segment between −1000 bp and −600 bp might have masked the activation of the TPP1 promoter (Fig. 4*A*). Subsequently, the region between −200 bp and −100 bp was found to bind transcription factors such as enhancer of zeste homolog 1 (EZH1), ESR1, DEC1, MYC, ZEB1, and MYB (Fig. 4*B*) through the online analysis tool hTFtarget (https://guolab.wchscu.cn/hTFtarget/#!/). Then, EZH1, ESR1, DEC1, MYC, ZEB1, and MYB were silenced by si-RNA in MKN-45 or HGC-27 cells, respectively (Supplementary Figs. S2 and S3). We found that lowest expressions of TPP1 were observed in EZH1-silenced MKN-45 or HGC-27 cells, indicating that the transcription of TPP1 was most significantly affected by EZH1 (Fig. 4*C*). Moreover, in both MKN-45 and HGC-27 cells, the mutation of the EZH1 binding site in the TPP1 promoter resulted in EZH1 being unable to effectively regulate the luciferase activity of the TPP1 promoter (−200, +15) (Fig. 4*D*). In addition, TPP1 was markedly downregulated in EZH1-kncokdown MKN-45 (decreased to 24.23%) or HGC-27 (decreased to 31.41%) cells, while TPP1 expressions were strikingly raised in EZH1-overexpressed MKN-45 (2.20 fold compared with control) or HGC-27 (2.73 fold compared with control) cells (Fig. 4*E*). According to ChIP-qPCR results (Fig. 4*F*), EZH1 might bind with the promoter of TPP1 at the P2 site shown in Figure 4*B*. In *H*. *pylori*-treated MKN-45 or HGC-27 cells, EZH1 and TPP1 expressions were visibly enhanced, which were remarkably decreased by silencing EZH1 (Fig. 4*G*). Additionally, silenced EZH1 also abrogates MKN-45 or HGC-27 cells invasion and migration (Supplementary Fig. S4). Cycloheximide (CHX)-treated MKN-45 or HGC-27 cells, EZH1 expressions were appreciably decreased as incubation duration increased from 1 h to 5 h. However, in the presence of *H*. *pylori*, the decline in EZH1 expression was markedly blocked in CHX-treated MKN-45 or HGC-27 cells as incubation duration increased from 1 h to 5 h (Fig. 4*H*), implying that *H*. *pylori* enhanced the stability of EZH1 in GC cells.

### TPP1 knockdown reversed the *H*. *pylori*-induced GC cell metastasis

**Figure 5:**
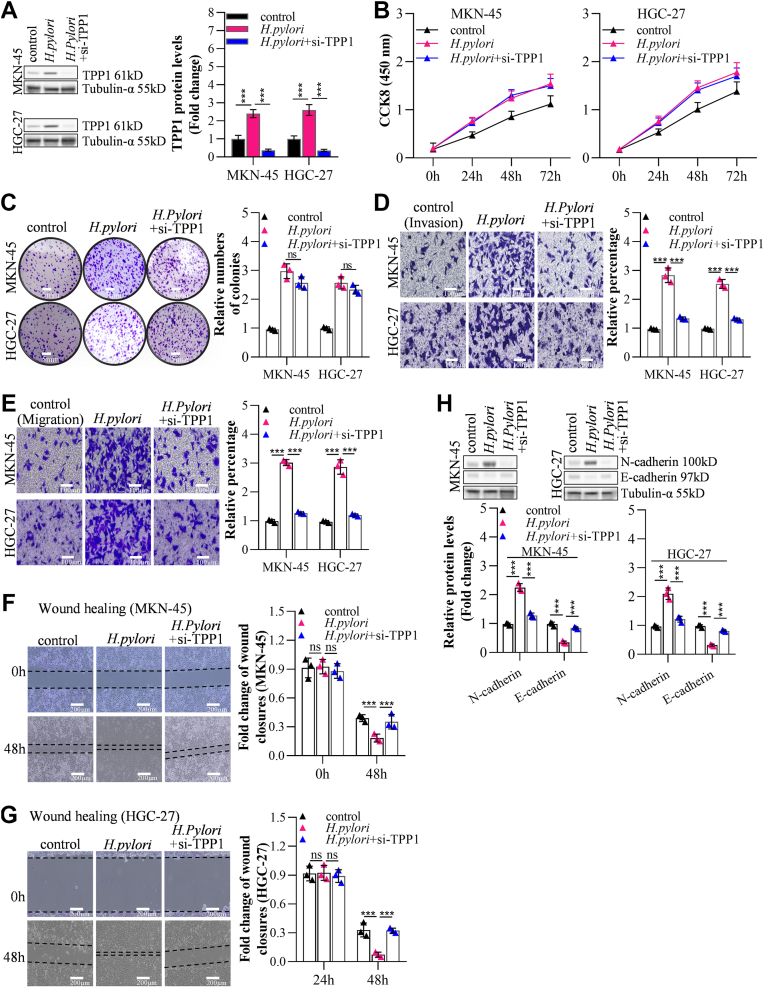
**TPP1 knockdown reversed the *H*. *pylori*-induced GC cell metastasis *in vitro***. *A*, the effect of TPP1 knockdown combined with *H*. *pylori* on TPP1 expression in GC cells was detected by Western blot. (*B*) The proliferation of GC cells affected by *H*. *pylori* could not be reversed by TPP1 knockdown, as detected by the CCK-8 assay. *C*, the proliferation of GC cells affected by *H*. *pylori* could not be reversed by TPP1 knockdown, as detected by the colony formation assay. *D* and *E*, The effects of TPP1 knockdown on reversing the influence of *H*. *pylori* on the invasion and migration of GC cells were detected by Transwell invasion and migration assays. *F* and *G*, the effect of TPP1 knockdown on reversing the influence of *H*. *pylori* on the migration of GC cells was detected by wound healing analysis. *H*, the effect of TPP1 knockdown on reversing the influence of *H*. *pylori* on the expression of invasion and migration markers N-cadherin and E-cadherin was detected by Western blot. (∗*p* < 0.05, ∗∗*p* < 0.01, ∗∗∗*p* < 0.001 indicate statistically significant differences. Ns indicate no significant difference).

In MKN-45 or HGC-27 cells, TPP1 expressions were sharply enhanced by *H*. *pylori*, which were substantially repressed by sh-TPP1 (Fig. 5*A*). Cell viabilities (by 1.37 and 1.28 fold in MKN-45 and HGC-27, respectively, at 72 h point) (Fig. 5*B*) and relative numbers of colonies (by 2.97 and 2.55 fold in MKN-45 and HGC-27, respectively) (Fig. 5*C*) were notably enhanced by *H*. *pylori*, which were mildly changed by silencing TPP1. *H*. *pylori* increased migrated (by 2.83 and 2.53 fold in MKN-45 and HGC-27, respectively) and invaded (by 3.01 and 2.87 fold in MKN-45 and HGC-27, respectively) GC cells considerably (Fig. 5, *D* and *E*), as well as noticeably diminished fold change of wound closures (by 52.99 and 77.78% in MKN-45 and HGC-27, respectively) (Fig. 5, *F* and *G*), which were substantially rescued by silencing TPP1. Additionally, strikingly upregulated N-cadherin and downregulated E-cadherin in *H*. *pylori*-stimulated MKN-45 or HGC-27 cells were remarkably reversed by silencing TPP1 (Fig. 5*H*).

### TPP1 enhanced ERS by disturbing the interaction between GPR78 and PERK

**Figure 6:**
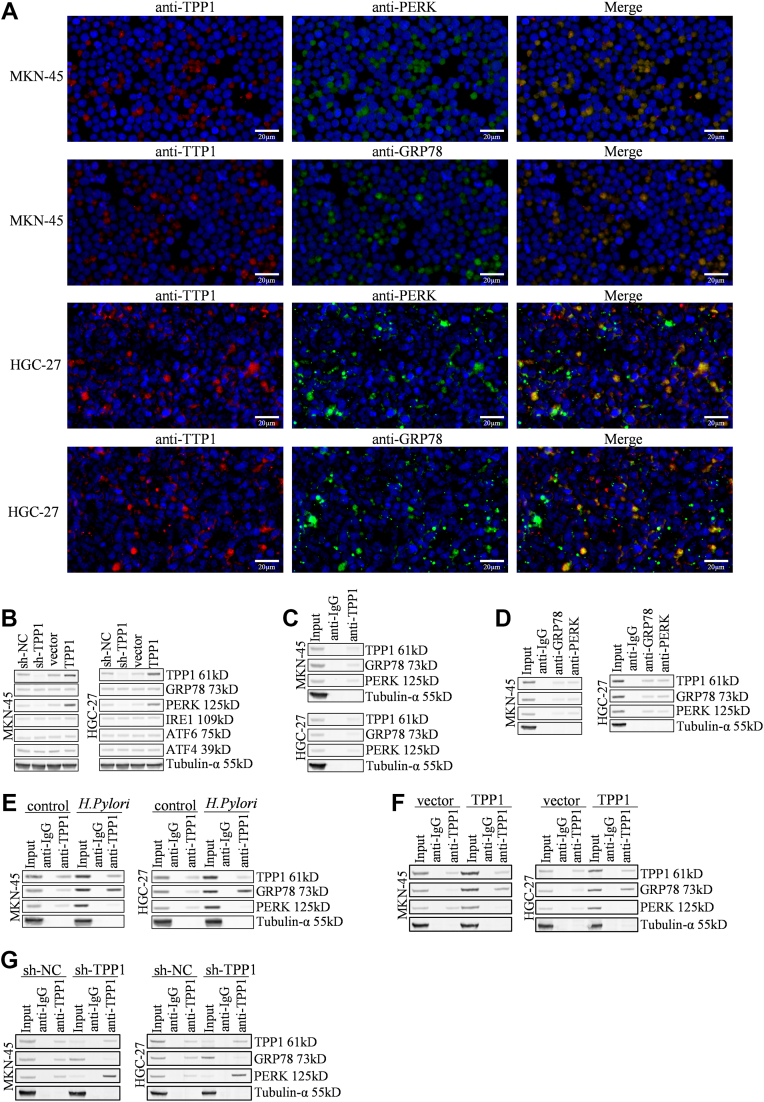
**TPP1 enhanced ERS by disturbing the interaction between GPR78 and PERK *in vitro***. *A*, the colocalization of TPP1 with GRP78/Bip and TPP1 with PERK was detected by immunofluorescence. (*B*) The knockdown of TPP1 in GC cells resulted in reduced PERK expressions, but not GRP78, IRE1, ATF4, and ATF6. *C*, endogenous IP results showed that TPP1 could interact with GRP78 and PERK. *D*, endogenous IP results showed that GRP78 and PERK could interact with TPP1. *E*, endogenous IP results showed that *H*. *pylori* could enhance the interaction between TPP1 and GRP78, leading to the separation of GRP78 from PERK. (*F*) Endogenous IP results showed that the overexpression of TPP1 could increase its interaction with GRP78, leading to the separation of GRP78 from PERK. *G*, endogenous IP results showed that the knockdown of TPP1 could weaken its interaction with GRP78. GRP78, glucose-regulated protein.

Subsequently, we checked influences of TPP1 on ERS in GC cells. Firstly, we found that TPP1 and GRP78, as well as TPP1 and PERK, were co-located in the cytoplasm of MKN-45 or HGC-27 cells (Fig. 6*A*). Following knocking down TPP1, only PERK was largely downregulated in MKN-45 or HGC-27 cells, with expressions of GRP78, IRE1, ATF4, and ATF6 nearly unchanged. Similar results were observed in TPP1-overexpressed MKN-45 or HGC-27 cells (Fig. 6*B*). According to IP experiments in MKN-45 or HGC-27 cells, interactions between TPP1 and GRP78, as well as TPP1 and PERK, were observed (Fg 6C), while GRP78 or PERK interacted with TPP1 in MKN-45 or HGC-27 cells (Fig. 6*D*). Moreover, the interaction between TPP1 and GRP78 was enhanced, while the interaction between TPP1 and PERK was restrained by *H*. *pylori* (Fig. 6*D*). Similarly, the interaction between TPP1 and GRP78 was enhanced, while the interaction between TPP1 and PERK was restrained by TPP1 overexpression (Fig. 6*E*). Oppositely, the interaction between TPP1 and GRP78 was reduced, while interaction between TPP1 and PERK was enhanced by silencing TPP1 (Fig. 6*F*).

### Blocking ERS overturned effects of TPP1 overexpression on GC cell metastasis

**Figure 7:**
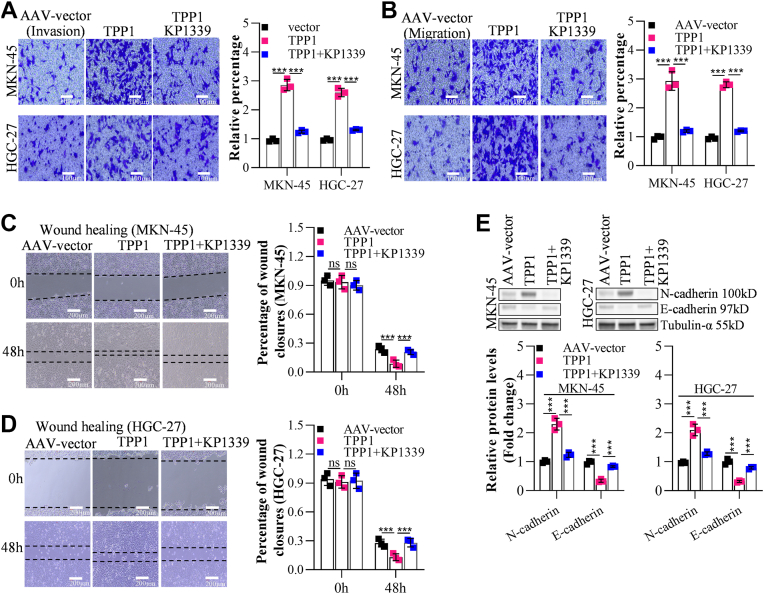
**Block ERS overturned effects of TPP1 overexpression on GC cell metastasis *in vitro***. *A* and *B*, transwell invasion and migration assays demonstrated that effects of TPP1 overexpression on the invasion and migration of GC cells could be reversed by KP1339. *C* and *D*, Wound healing analysis showed that effects of TPP1 overexpression on the migration of GC cells could be reversed by KP1339. *E*, western blot analysis revealed that effects of TPP1 overexpression on the expression of the invasion and migration markers N-cadherin and E-cadherin could be reversed by KP1339. (∗*p* < 0.05, ∗∗*p* < 0.01, ∗∗∗*p* < 0.001 indicate statistically significant differences. Ns indicate no significant difference).

To validate that TPP1 influenced GC cell migration by activating ERS, MKN-45 or HGC-27 cells were transfected with AAV-TPP1 for 72 h, followed by treatment with KP1339, an inhibitor of ERS. Numbers of migrated (by 2.86 and 2.62 fold in MKN-45 and HGC-27, respectively) and invaded (by 2.93 and 2.8 fold in MKN-45 and HGC-27, respectively) cells were markedly raised by TPP1 overexpression (Fig. 7, *A* and *B*), along with sharply decreased fold change of wound closures (by 63.78 and 53.01% in MKN-45 and HGC-27, respectively) (Fig. 7, *C* and *D*), which were considerably rescued by KP1339. Moreover, enhanced N-cadherin expression and decreased E-cadherin expressions in TPP1-overexpressed MKN-45 or HGC-27 cells were perceptibly reversed by KP1339 (Fig. 7*E*).

### TPP1 knockdown repressed GC cell metastasis in xenograft nude mice model

AAV-sh-TPP1 and AAV-sh-NC transfected GC cells were intravenously injected into nude mice, respectively. In liver, lymphatic, and lung tissues, tissue metastasis incidences were strikingly reduced in AAV-sh-TPP1 transfected MKN-45 or HGC-27 cell xenograft model comparing to AAV-sh-NC (Fig. S1, *A*–*C*), hinting that *in vivo* GC cell metastasis was noticeably inhibited by silencing TPP1.

## Discussion

TPP1, as a core component of the telomere complex, performs a critical function in tumor developments. It has been demonstrated that TPP1 is abnormally overexpressed in a variety of malignant tumors and promotes tumor cell migration by regulating telomere stability, DNA damage repair, and the activity of signaling pathways. For example, in esophageal cancer, the expression of TPP1 is sharply upregulated and its knockdown can markedly reduce the migration ability of tumor cells by causing cell cycle arrest at the G1 phase (25). Harmonious with data claimed by Huang (26), TPP1 was found noticeably upregulated in both GC tissues and GC cell lines, expression levels of which were correlated with poor prognosis. Furthermore, TPP1 perceptibly enhanced the migration and invasion of GC cells without impacting the proliferation ability, suggesting that TPP1 was a critical metastasis-related factor involved in GC development. EMT is considered as a crucial process for tumor cells to gain invasive and migratory abilities. Its core characteristics, including the loss of E-cadherin and the ectopic expression of N-cadherin, are referred to as the "cadherin switch" (29, 30). E-cadherin, regarded as the key protein for intercellular adhesion junctions in epithelial cells, is connected to the actin cytoskeleton through β-catenin, thus maintaining the stability of epithelial tissues (31). During the process of EMT, the expression of E-cadherin is inhibited by transcriptional repressors (such as Snail, ZEB1) through epigenetic modifications or direct binding to the CDH1 promoter, resulting in the loss of intercellular adhesion, the release of β-catenin, and the activation of the Wnt signaling pathway, which promotes the mesenchymal phenotypic transformation (32). Meanwhile, the up-regulation of N-cadherin expression enhances cell motility through the activation of signaling pathways such as FGFR/ERK, promoting tumor stromal infiltration and distant metastasis (33). Here, EMT progression in GC cells was strikingly enhanced by TPP1, indicating that TPP1 might facilitate GC cell migration and invasion *via* activating the EMT progression. Additionally, *H*. *pylori* triggered the upregulation of TPP1, along with enhanced proliferation and migration ability, and activated EMT progression. However, TPP1 knockdown noticeably reversed influences of *H*. *pylori* on the migration and EMT progression of GC cells, but not proliferation, implying that TPP1 participated in the *H*. *pylori*-induced metastasis in GC cells possibly by enhancing the EMT progression.

Through series of experimental screening, EZH1 was found to be engaged in mainly regulating the transcription of TPP1 in GC cells. EZH1, as one of the core catalytic subunits of polycomb repressive complex 2, together with EZH2, mediates the trimethylation of histone H3 at lysine 27 (H3K27me3), thereby achieving transcriptional regulation of target genes through epigenetic regulation (34). The C-terminus of EZH1 contains a unique cysteine-rich domain, the distribution pattern of whose cysteine residues is highly conserved in evolution and may be involved in the stabilization and maintenance of heterochromatin (35). In tumors, EZH1 is able to partially compensate for the function of EZH2, maintaining the methyltransferase activity of PRC2, and thereby regulating the plasticity and heterogeneity of tumor cells (36). In addition, EZH1 regulates the expression of EMT-related genes by maintaining specific chromatin accessibility states, thereby enhancing the invasiveness and metastatic potential of tumor cells (36). In GC, the overexpression of EZH1 is associated with tumor progression and poor prognosis. TRIM21 enhances the therapeutic effect of Apatinib on gastric cancer by inhibiting the stability of EZH1 (37). Herein, EZH1 was found dramatically influenced the transcription of TPP1 on the specific binding site (P2, Fig. 4*B*), and silencing EZH1 abolished *H*. *pylori*-induced upregulation of TPP1 in GC cells, indicating that TPP1 was positively transcriptionally regulated by EZH1 in GC cells. CHX is widely used in the study of protein expression regulation and is currently employed as a translation inhibitor, which mainly functions by inhibiting the elongation process of eukaryotic ribosomes, thereby blocking the synthesis of nascent proteins (38). Here, the stability of EZH1 in the presence of CHX was enhanced by *H*. *pylori*, implying that *H*. *pylori* might control the translation process of EZH1 to upregulate its expression in GC cells.

ERS is an adaptive response triggered by cells under stress such as hypoxia, nutrient deprivation, or accumulation of misfolded proteins. The core mechanism is to restore ER homeostasis through the UPR (39). In ERS, the interaction between GRP78 and PERK is the core mechanism for regulating the UPR. Under normal conditions, GRP78 acts as a molecular chaperone and binds to transmembrane sensors like PERK, inhibiting their activity (40). When unfolded proteins accumulate in the ER, GRP78 dissociates and preferentially binds to the abnormal proteins. This leads to the autophosphorylation and activation of PERK. PERK then reduces global protein synthesis through eIF2α, and selectively activates ATF4 and its downstream pro-apoptotic factor CHOP, which coordinates the decision of cell survival or apoptosis, resulting in the occurrence of ERS (41, 42). Here, TPP1 was found to competing with PERK to interact with GRP78, resulting in the separation of GRP78 from PERK, which further led to homopolymerization and autophosphorylation of PERK domain to induce ERS. Furthermore, *H*. *Pylori* enhanced the binding between TPP1 and GRP78, indicating that *H*. *Pylori* activated TPP1/GRP78 axis to trigger ERS in GC cells. Moreover, influences of TPP1 overexpression on the migration and invasion of GC cells was overturned by KP1339, an inhibitor of GRP78. *In vivo* data confirmed that the metastasis of GC cells was repressed by silencing TPP1, which validated that TPP1 activated GRP78-mediated ERS to facilitate the GC cell metastasis.

There were several limitations in the present study. Firstly, we found that *H*. *pylori* might stabilize the translation process of EZH1 to upregulate its expression in GC cells. However, the mechanism how *H*. *pylori* enhanced the stability and translation of EZH1 remains to be further studied. Additionally, the mechanism that TPP1 activated GRP78-mediated ERS to facilitate GC cell metastasis will be further identified in animal studies by treating TPP1-overexpressed GC cell xenograft model with KP1339.

Taken together, TPP1, induced by *H*. *pylori*, promoted GC cell metastasis *via* enhancing ERS by interacting with GRP78, which might be potential novel molecular targets for treating GC in the clinic.

## Experimental procedures

### Starbase analysis

Expressions of TPP1 in 375 tumor samples and 32 normal samples in stomach adenocarcinoma were analyzed in the data base of Starbase (https://rnasysu.com/encori/).

### Human ethics approval

Sixty GC patients treated in our hospital were included, followed by collecting adjacent tissues and GC tissues. All experiments involving human samples were conducted in accordance with the relevant guidelines and regulations and were approved by the Ethics Committee of Jiangyin People's Hospital (approve no.: JYYYEC78–1/March 1, 2022) and consents were obtained from the patients. The human studies reported in this article abide by the principles of the Declaration of Helsinki.

### RT-qPCR assay

Total RNA isolation from both tissue samples and cultured cells was carried out with Trizol solution following standard protocols. Complimentary DNA synthesis was then performed through enzymatic conversion utilizing a commercial cDNA synthesis kit (6210A, TaKaRa) according to manufacturer's guidelines. Quantitative analysis was subsequently conducted with SYBR Green-based real-time PCR reagents (204,243, QIAGEN) from the same manufacturer for precise measurement of transcriptional activity. Amplification reactions were executed on the ABI 7300 Real-Time PCR platform (ABI 7300, Applied Biosystems) with standardized thermal cycling parameters. For data normalization, Tubulin-α expression served as the constitutive internal reference. Final quantification of mRNA abundance was calculated through the 2^−ΔΔCT^ calculation approach for relative expression determination.

### Western blot analysis

For protein isolation, cellular or tissue specimens underwent mechanical disruption in lysis buffer followed by concentration normalization using bicinchoninic acid assay. Equalized lysates were mixed with Laemmli sample buffer (4:1 v/v) and thermally denatured through boiling at 100 °C for 5 min with subsequent rapid cooling in an ice bath. Electrophoretic separation was conducted on precast 10% Tris-glycine gels under reducing conditions at 120 V constant voltage. Semi-dry transfer equipment (Trans-Blot SD Semi-Dry Transfer Cell, Bio-Rad) facilitated protein migration onto PVDF membranes (0.45 μm pore size) using 25 V transfer buffer (pH 8.3) for 45 min. Membranes underwent sequential processing: initial 1 h blocking in TBS-T containing 5% non-fat dried milk, followed by 16 h incubation at 4°C with species-specific primary antibodies (anti-TPP1 (14,667), N-cadherin (13,116), E-cadherin (3195), EZH1 (42,088), GRP78 (3177), PERK (3192), IRE1 (3294), ATF4 (11,815), ATF6 (65,880) [1:1000]; α-tubulin (2144) [1:4000]; CST). After 3 10-min TBST washes, membranes received secondary antibodies (1:2000, 58,802, CST) for 60 min at ambient temperature. Chemiluminescent detection employed freshly prepared ECL substrate with 60 s membrane exposure in dark conditions. Digital imaging was captured using ChemiDoc MP Imaging System (ChemiDoc MP, Bio-Rad) and densitometric analysis performed through ImageLab (https://www.bio-rad.com/zh-cn/product/image-lab-software?ID=KRE6P5E8Z) software (v6.1) with background subtraction normalization.

### Kaplan-Meier (KM) plotter analysis

To explore the relationship between individual TPP1 mRNA expression levels and survival outcomes in GC, we used the KM plotter database (http://kmplot.com/analysis). The findings were presented with key statistical metrics, including the hazard ratio, 95% confidence intervals, and the computed log rank *p*-value, which collectively provided insights into the prognostic significance of TPP1 mRNA expression in GC.

### Cell lines and treatments

Human gastric mucosa epithelial cells (GES-1 cells, A01X717) and GC cell lines (MKN-45 (A01X857), HGC-27 (A01X758), BGC-823 (AXB10159), MGC-803 (A01X852), and SNU-1 cells (A01X968)) were achieved from Shanghai Fusheng Industrial Co., Ltd with STR profiling and mycoplasma-free reports, and cultured in DMEM with 10% FBS under 5% CO_2_ and 37 °C. To obtain TPP1-knockdown GC cells, cells were transfected with adeno-associated virus (AAV) containing a TPP1-targeting shRNA (AAV-sh-TPP1, sequence: 5′-CCGGGCTAGCTAGCTAGCTAGCTAGCTCGAGCTAGCTAGCTAGCTAGCTTTTTG-3′) for 72 h, with AAV-sh-NC (sequence: 5′-CCGGGCTAGCTAGCTAGCTAGCTAGCTCGAGCTAGCTAGCTAGCTAGCTTTTTG-3′) as the negative control. To achieve TPP1-overexpressed GC cells, cells were transfected with AAV containing the TPP1-overexpressed vector (AAV-TPP1) for 72 h, with AAV-vector as the negative control. For TPP1 promoter binding transcriptional factors, the siRNAs were transfected as our group previous reported (43), and the sequences were listed in Supplementary Table S1.

### CCK-8 assay

The cells were seeded into a 96-well plate after transfection. Then, the DMEM culture medium with 10 g/dl CCK - eight reagent was loaded, and the cells were treated for 0 h, 24 h, 48 h, and 72 h. The OD_450_ nm was checked with a microplate reader (SpectraMax i3x,), and the proliferation curve was plotted.

### Clone formation assay

Cells in the logarithmic growth phase were taken and digested with trypsin. Then, they were seeded in a 6 - well plate (200 cells/well). After being cultured until visible clones appeared, cells were collected. Then, they were fixed with 4 g/dl paraformaldehyde for 20 min and stained with 0.1 g/dl crystal violet for 30 min. Finally, cells were observed, photographed (Axiovert 40, Zeiss), and analyzed.

### Migration assay by transwell

Cells were resuspended in serum-free DMEM medium and added to the upper chamber, while 600 μl of complete DMEM medium containing 10% FBS was added to the lower chamber. After 24-h incubation, the chamber was removed and the medium in the upper chamber was aspirated. Cells were fixed with 4% paraformaldehyde for 30 min and stained with 0.05% crystal violet for 30 min. Residual cells in the upper chamber were carefully removed using a moist cotton swab. The membrane was air-dried and examined under an inverted optical microscope (Axiovert 40, Zeiss,) for cell counting.

### Invasion assay by transwell

The Matrigel matrix was pre-coated at the center of the upper chamber membrane. Complete medium containing 10% FBS (600 μl) was added to the lower chamber, and cell suspension was loaded into the upper chamber. Subsequent procedures were performed following the same protocol as described for the migration assay, including fixation, staining, cell removal, drying, and microscopic observation (Axiovert 40, Zeiss).

### Wound healing assay

Cells were seeded into 6-well plates, with five equidistant parallel lines pre-marked on the plate bottom for imaging localization. After reaching monolayer confluence, the medium was replaced with serum-free DMEM for 24-h serum deprivation synchronization. A vertical wound line perpendicular to the pre-marked lines was created using a 200 μl pipette tip, followed by three PBS washes to remove dislodged cells. Group-specific mixed medium (2 ml/well) was then added as previously described. At 48 h post-treatment, the wound areas were observed and photographed under an inverted phase-contrast microscope (Axiovert 40, Zeiss). The wound area was quantified using Image-Pro Plus (https://mediacy.com/image-pro/) software, and the fold change of wound closure was calculated.

### Detection of luciferase activities

The luciferase activity of different segments within the TPP1 promoter was analyzed using the pGL3-Basic plasmid system and associated kits (E1751, Promega). All experimental protocols were rigorously executed according to the manufacturer's guidelines.

### ChIP-qPCR assay

Cells were first cross-linked with 1% formaldehyde and incubated for 10 min, after which glycine was added to terminate the reaction. Cells were then washed with pre-cooled PBS, lysed with lysis buffer, and sonicated to fragment the chromatin. The lysates were incubated with anti-EZH1 antibody (42,088, CST) overnight, followed by the addition of Protein A agarose beads for incubation and washing of beads. Finally, the complexes were eluted, cross-links were reversed, and DNA was purified. RT-PCR analysis was performed using primers targeting the TPP1 promoter region at binding sites of P1, P2, P3, and P4 shown in Figure 4*B*.

### Immunoprecipitation (IP) assay

An experiment was conducted on GC cells to assess the interaction between TPP1 and GRP78, or TPP1 and PERK through an IP assay. The cells were first lysed using ice-cold lysis buffer. After centrifugation of the lysates, the supernatants were incubated with –the indicated antibodies at 4 °C overnight for IP. Subsequently, all the samples were added with Protein G beads and they were incubated at 4 °C for 2 h. Beads were then washed with the above lysis buffer. Next, they were heated at 98 °C for 10 min after adding 4 × loading buffer. At last, the beads were pelleted by centrifugation and discarded, while the final immunoprecipitated samples were collected. These collected samples were then processed and used for Western blot as described above.

### Immunofluorescence (IF) staining

IF assays were performed as our previous report briefly (44) to confirm the expression and localization of proteins, including TPP1, PERK, GRP78, in MKN-45 and HGC-27 cells. Cells were planted in a 12–well plate for 24 h. Then, fixed in 4% paraformaldehyde for half an hour following with washed 3 times with cold PBS at 4 °C. Cells were incubated overnight at 4 °C with anti–TPP1 (NBP1-31758, Novus Biologicals), anti–PERK (NBP1-80930, Novus Biologicals), anti-GRP78 (NBP1-06274, Novus Biologicals). After washing with PBS, the cells were incubated with DyLight633 or DyLight488 (35,563 or 35,553, Thermo Fisher Scientific) for an hour. Meanwhile, the cell nuclei were stained with Hoechst (C1011, Beyotime) for 10 min. Finally, they were washed with PBS, and these stained cells were observed under a Zeiss fluorescence microscope (Carl Zeiss AG, Oberkochen).

### Xenograft model in nude mice

Female nude mice (6–9 weeks) were purchased from GemPharmatech Co. Ltd and divided into two groups (n = 6/group). AAV-sh-TPP1 and AAV-sh-NC transfected GC cells were intravenously injected into nude mice *via* tail vein, respectively. Two months later, lungs, lymph nodes, and livers were collected from mice and subjected to H&E staining to observe the metastasis of tumor cells in these organs. All rodent experiments were approved by the Ethics Committee of Jiangyin People's Hospital (approve no.: JYYYEC96–4/June 1, 2024). Experiments were terminated immediately when mice met any of the criteria listed below (1), Body weight changes: the mouse lost >15% of its initial body weight within 1 week, or showed continuous weight loss for 3 days without recovery (2). Behavioral and mental status: marked reduction in activity; inability to stand/walk normally; absence of food/water intake for >24 h; or signs of distress (3). Organ dysfunction symptoms: mice presented with abnormal respiration, pale/cyanotic mucous membranes, delayed tail blood refill, or extensive skin rashes/ulcers.

### H&E staining

After tumor tissue samples were fixed in 4% neutral formaldehyde solution for 24 h, they were rinsed in running water for 1 h to remove residual formaldehyde. Then, the samples were transferred to 70% ethanol solution for dehydration, with the solution changed every 15 min for 3 times. This was followed by dehydration in 80%, 90%, 95%, and 100% ethanol solutions, each for 15 min. Next, samples were immersed in xylene for 15 min twice for clearing. After that, samples were embedded in paraffin to form paraffin blocks. Sections of 4 microns thickness were cut and placed on glass slides, then incubated in a 60 °C oven for 1 h. After incubation, sections were immersed in xylene for 15 min twice for dewaxing, and then rehydrated through graded ethanol solutions (100%, 95%, 90%, 80%, 70%) for 15 min each. Subsequently, sections were stained with hematoxylin staining solution for 5 min and rinsed with water. Then, the sections were differentiated with 1% hydrochloric acid alcohol for 30 s and rinsed with water. After that, sections were stained with eosin staining solution for 2 min and rinsed with water. Finally, sections were sealed with neutral gum and observed under a microscope (Axiovert 40, Zeiss,).

### Statistical analysis

Data underwent statistical analysis utilizing GraphPad Prism (https://www.graphpad.com/features) software, version 8.0, with results presented as the mean ± standard deviation. For comparisons between two groups, a two -tailed *t* test was utilized, whereas ANOVA was implemented to assess differences among 3 or more groups. Statistical significance was determined by a *p*-value less than 0.05.

## Ethics approval and consent to participate

All rodent experiments (approve no.: JYYYEC96–4/June 1, 2024) and clinical tissues collection (approve no.: JYYYEC78-1March 1, 2022) were approved by the Ethics Committee of Jiangyin People's Hospital. The consents were obtained from the patients whose tissues were collected for this research.

## Data availability

All data are contained within the manuscript. Raw data are available upon request.

## Supporting information

This article contains supporting information.

## Conflict of interests

The authors declare that they have no conflicts of interest with the contents of this article.
