# Supplementary material for: Telomere-protecting protein 1 promotes gastric cancer cell metastasis via enhancing endoplasmic reticulum stress
Source: J Biol Chem. 2025 Dec 1;302(1):110998. doi: 10.1016/j.jbc.2025.110998 (PMC12795675; doi:10.1016/j.jbc.2025.110998)
Supplement: Supplementary Material 1 [file mmc1.docx]

**Supplementary Figure S1**


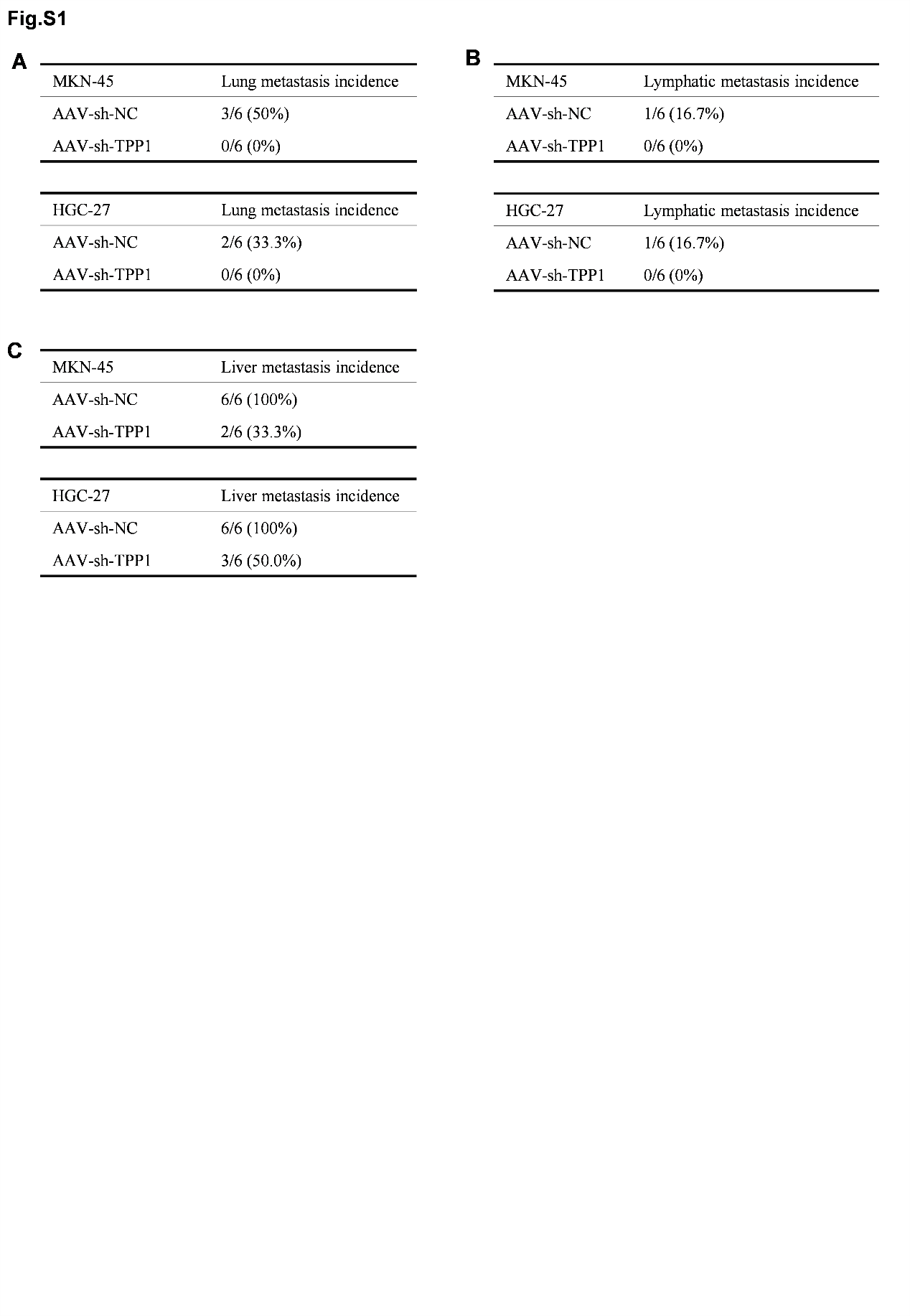


**Supplementary Figure S2**


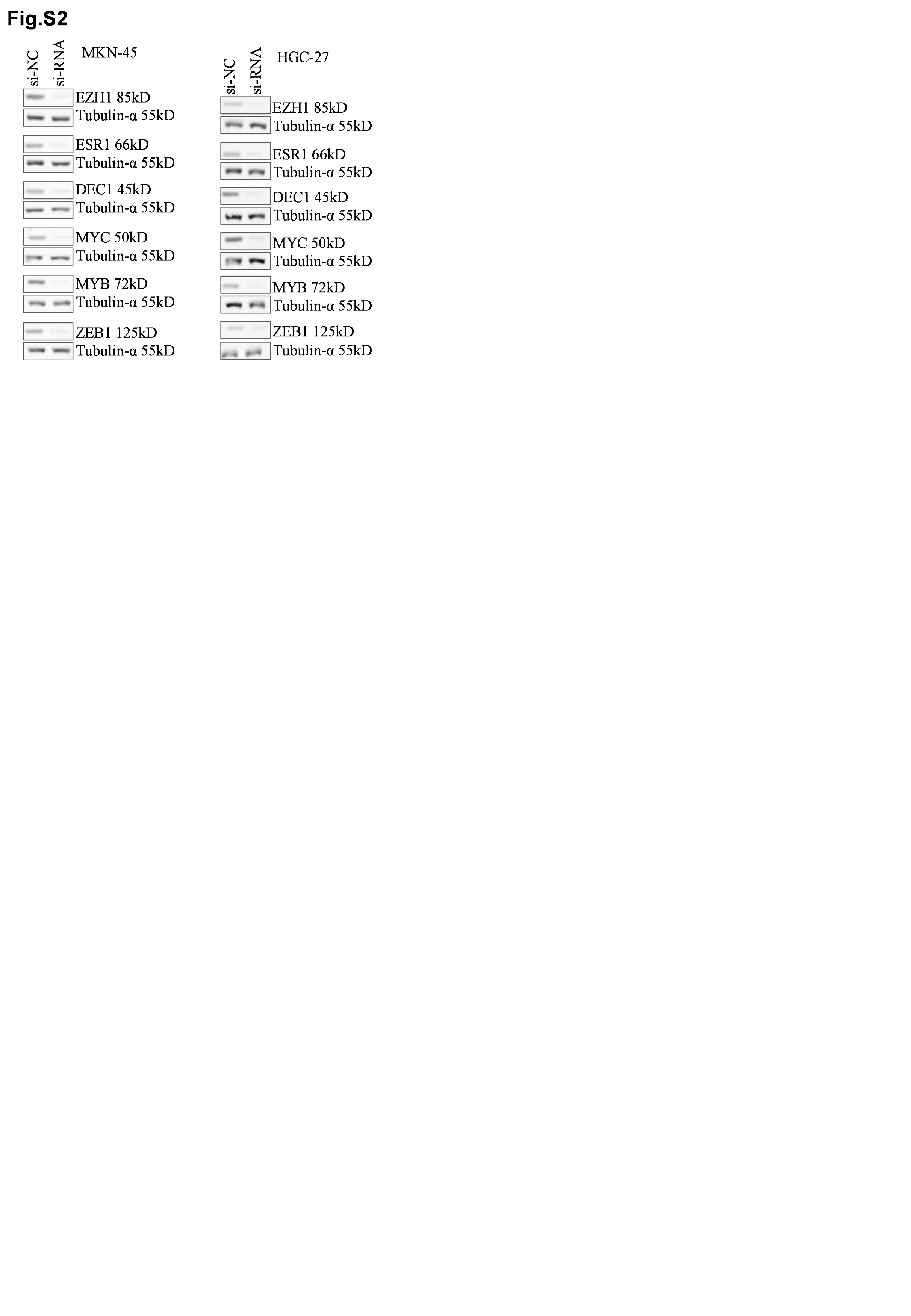


**Supplementary Figure S3**


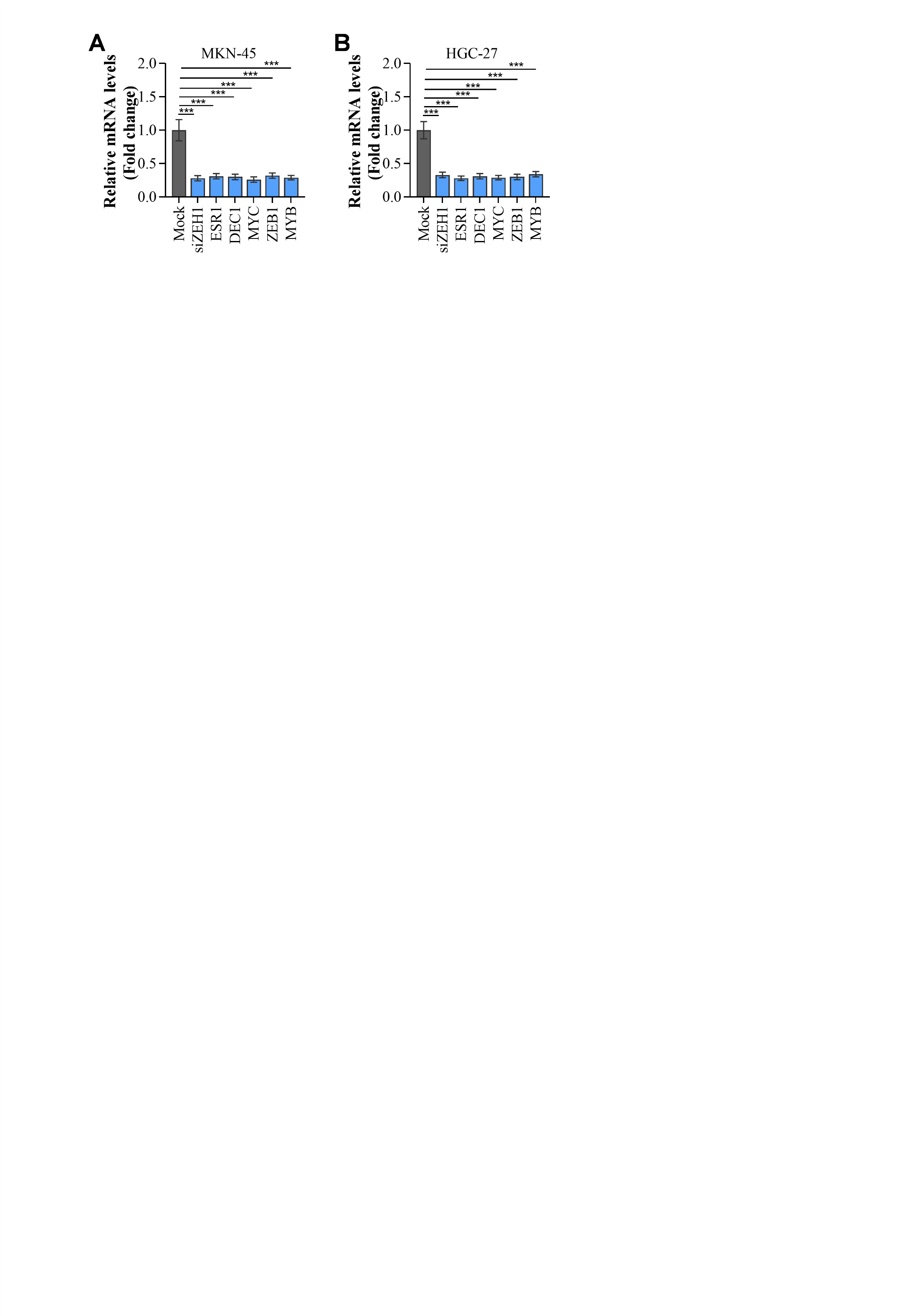


**Supplementary Figure S4**


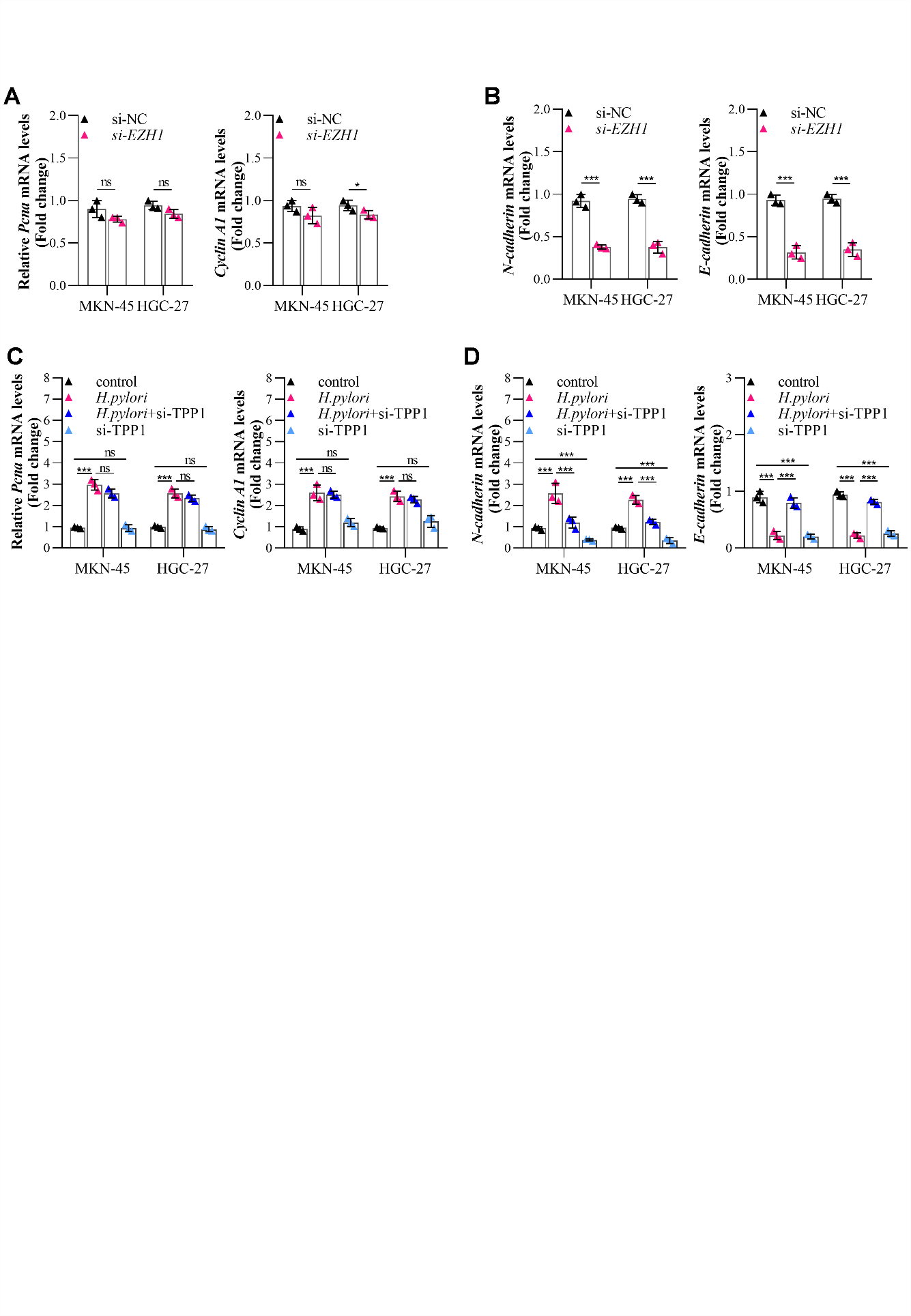


**Supplementary Table S1**: The used siRNAs in this study.

| Gene name | siRNAs (5’-3’) |
| --- | --- |
| EZH1 | #1: AAAUUUGCCACAUACAAAGCC dUdU |
|  | #2: UACACUUUUUGAGAAAAGGGU dUdU |
|  | #3: AUCUUCUACCAUAAAGUUCUG dUdU |
| ESR1 | #1: UCGAUUAUCUGAAUUUGGCCU dUdU |
|  | #2: AAUACUUCUCUUGAAGAAGGC dUdU |
|  | #3: AAGCAAAAUAAUAGAUUUGAG dUdU |
| DEC1 | #1: UUGUAAGUUUGAGAUGUUCGG dUdU |
|  | #2: AAGUUGUAAGUUUGAGAUGUU dUdU |
|  | #3: UAAUUGCGCCGAUCCUUUCUC dUdU |
| MYC | #1: AACAACAUCGAUUUCUUCCUC dUdU |
|  | #2: UUUUUUAAGGAUAACUACCUU dUdU |
|  | #3: UUCAAGUUUGUGUUUCAACUG dUdU |
| ZEB1 | #1: UGGUUUUGCUCAUUUUCUGCA dUdU |
|  | #2: AGGUAAAGCGUUUAUAGCCUC dUdU |
|  | #3: UUGAAUUUACGAUUACACCCA dUdU |
| MYB | #1: AUAAUUGGCAAUAACUUUCCA dUdU |
|  | #2: UCCAAUUCUCCCCUUUAAGUG dUdU |
|  | #3: ACUAUAUUUACAUGUAACGCU dUdU |
| Mock | UUCUCCGAACGUGUCACGUTT dUdU |
